# The actin-bundling protein Fascin-1 modulates ciliary signalling

**DOI:** 10.1093/jmcb/mjad022

**Published:** 2023-04-04

**Authors:** Lena Brücker, Stefanie Kornelia Becker, Vanessa Maissl, Gregory Harms, Maddy Parsons, Helen Louise May-Simera

**Affiliations:** Cilia Cell Biology, Institute of Molecular Physiology, Johannes Gutenberg University Mainz, 55128 Mainz, Germany; Cilia Cell Biology, Institute of Molecular Physiology, Johannes Gutenberg University Mainz, 55128 Mainz, Germany; Cilia Cell Biology, Institute of Molecular Physiology, Johannes Gutenberg University Mainz, 55128 Mainz, Germany; Imaging Core Facility, Cell Biology Unit, University Medical Centre, Johannes Gutenberg University Mainz, 55101 Mainz, Germany; Randall Centre for Cell and Molecular Biophysics, King's College London, London SE1 1UL, UK; Cilia Cell Biology, Institute of Molecular Physiology, Johannes Gutenberg University Mainz, 55128 Mainz, Germany

**Keywords:** cilia, actin, Wnt, signalling, ciliopathy, Bardet–Biedl syndrome, filopodia

## Abstract

Primary cilia are microtubule-based cell organelles important for cellular communication. Since they are involved in the regulation of numerous signalling pathways, defects in cilia development or function are associated with genetic disorders, collectively called ciliopathies. Besides their ciliary functions, recent research has shown that several ciliary proteins are involved in the coordination of the actin cytoskeleton. Although ciliary and actin phenotypes are related, the exact nature of their interconnection remains incompletely understood. Here, we show that the protein BBS6, associated with the ciliopathy Bardet–Biedl syndrome, cooperates with the actin-bundling protein Fascin-1 in regulating filopodia and ciliary signalling. We found that loss of *Bbs6* affects filopodia length potentially via attenuated interaction with Fascin-1. Conversely, loss of *Fascin-1* leads to a ciliary phenotype, subsequently affecting ciliary Wnt signalling, possibly in collaboration with BBS6. Our data shed light on how ciliary proteins are involved in actin regulations and provide new insight into the involvement of the actin regulator Fascin-1 in ciliogenesis and cilia-associated signalling. Advancing our knowledge of the complex regulations between primary cilia and actin dynamics is important to understand the pathogenic consequences of ciliopathies.

## Introduction

Primary cilia are microtubule-based sensory cell protrusions vital for cell homeostasis and tissue development. They act as sensory antennae, receiving and transducing cues related to cellular signalling pathways such as sonic hedgehog, platelet-derived growth factor, and Wnt (Goetz and Anderson, 2010; Wallingford and Mitchell, 2011; May-Simera and Kelley, 2012; Lee, 2020). Thus, defects in primary cilia or ciliary proteins are known to be associated with a group of genetic disorders, so-called ciliopathies (Reiter and Leroux, 2017; Chen et al., 2021). Although primary cilia predominantly coordinate the cellular microtubule network, ciliary defects also disrupt the regulation of the actin cytoskeleton (Brücker et al., 2020; Smith et al., 2020). Conversely, actin polymerization is a negative regulator of ciliogenesis (Bershteyn et al., 2010; Kim et al., 2010, 2015; Liang et al., 2016). The interconnected phenotype between primary cilia and actin dynamics is complex and not fully understood, and thus any new insights into these processes would have wide-reaching consequences.

Numerous ciliary proteins have already been shown to coordinate the actin cytoskeleton, emphasizing the interplay between cilia and actin (Yin et al., 2009; Kim et al., 2010; May-Simera et al., 2016). An important subset of ciliary proteins involved in actin dynamics includes the Bardet–Biedl syndrome (BBS) proteins, defects in which are associated with the archetypical ciliopathy BBS. Besides their classically defined functions in cilia development, maintenance, and trafficking (Wei et al., 2012; Nozaki et al., 2019; Patnaik et al., 2019), BBS proteins have been shown to be associated with downstream actin perturbations (Novas et al., 2015). Loss of *Bbs4, Bbs6, Bbs8*, and *Bbs15*, which exert different ciliary functions, results in defective actin-based cell migration and a disorganization of the actin cytoskeleton (Cui et al., 2013; Hernandez-Hernandez et al., 2013). This is associated with the upregulation of downstream RhoA signalling, resulting in increased numbers of focal adhesions. In zebrafish, bbs8 was further found to be required for the migration of neural crest cells and fibroblasts, and its loss was accompanied by a lack of polymerization of the actin cytoskeleton and disorganized microfilaments (Tobin et al., 2008). However, the direct link between ciliary BBS proteins and the actin cytoskeleton is still unclear.

Many actin phenotypes caused by ciliary defects thus far could be ascribed to aberrant non-canonical Wnt signalling, also referred to as planar cell polarity (PCP) signalling, a pathway known to affect downstream actin networks (Gerdes et al., 2007; Corbit et al., 2008; May-Simera et al., 2010, 2015; Cui et al., 2013; McMurray et al., 2013; Balmer et al., 2015; Wang et al., 2017). Upon activation of this pathway by Wnt ligands binding to the Frizzled receptor, the ciliary PCP protein Inversin recruits Dishevelled to the plasma membrane (Simons et al., 2005). Dishevelled in turn binds to formins and small GTPases, subsequently activating downstream actin regulators such as RhoA, Rock, and Jnk (Habas et al., 2001; Liu et al., 2008). Thus, Inversin acts as a key player in the switch from canonical to non-canonical Wnt signalling, inhibiting canonical Wnt and promoting directional cell migration via regulating Rho GTPases and the downstream actin network (Simons et al., 2005; Lienkamp et al., 2012; Veland et al., 2013; Werner et al., 2013). The non-canonical Wnt/PCP signalling also results in the development of actin-based filopodia that further distribute the Wnt signal to recipient cells (Stanganello et al., 2015; Mattes et al., 2018; Rosenbauer et al., 2020). Recent data indicate that BBS2, BBS6, and BBS8 interact with Inversin, facilitating its transport to the base of the cilium and enabling its function in non-canonical Wnt/PCP signalling (May-Simera et al., 2018; Patnaik et al., 2019). Consistently, knockout of *bbs6* and *bbs8* in zebrafish leads to enhanced canonical Wnt signalling and a loss of non-canonical Wnt/PCP signalling, supporting the cooperation between Inversin and BBS proteins (Ross et al., 2005; Gerdes et al., 2007; May-Simera et al., 2010, 2015).

Besides the reciprocity between ciliary proteins and Wnt signalling in regulating actin dynamics, it is plausible that there is a more direct connection between cilia and the actin cytoskeleton. Many types of proteins have been reported to participate in directly regulating the actin cytoskeleton, enabling cell migration, trafficking, and morphology changes. Since primary cilia are microtubule-based organelles, actin-binding proteins that also affect the microtubule network are of particular interest. Prominent examples for this include microtubule–actin cross-linking factor (MACF1), the inverted formin 1 (FHDC1), and Fascin-1, an actin-bundling protein important for filopodia formation (Wu et al., 2008; Young et al., 2008; Thurston et al., 2012; Antonellis et al., 2014; Villari et al., 2015). For MACF1 and FHDC1, a role in ciliogenesis has already been described (May-Simera et al., 2016; Copeland et al., 2018); however, a functional link between Fascin-1 and ciliary proteins has not been investigated thus far.

In the current work, we shed light on the functional regulation between ciliary proteins and actin regulators. We found that loss of *Bbs6* affects filopodia length potentially via attenuated interaction with the filopodia regulator Fascin-1. Interestingly, knockdown of *Fascin-1* affected cilia number, suggesting a role of Fascin-1 in initiating ciliogenesis. Furthermore, we found that Fascin-1 cooperates with BBS6 in regulating ciliary Wnt signalling via downstream Wnt targets such as Cyclin D1. Taken together, our results demonstrate a role for Fascin-1 in bridging the regulation of primary cilia and actin networks, connecting phenotypes in both ciliogenesis and actin via coordination of signalling pathways such as Wnt.

## Results

### BBS6 regulates filopodia stability via the interaction with Fascin-1

Loss of ciliary proteins BBS6 and BBS8 was previously shown to be associated with defects in cell migration and disrupted actin networks (Tobin et al., 2008; Hernandez-Hernandez et al., 2013). To further elucidate this association, we analyzed filopodia in mouse embryonic fibroblasts (MEFs). Fibroblasts represent an excellent model to study the interplay between cilia and actin-dependent cell migration, since they are able to develop primary cilia and, being derived from connective tissues, have a robust migratory phenotype. Since mice recapitulate human ciliopathy phenotype really well (Kretschmer et al., 2019; Schneider et al., 2021), this mouse-derived cell model is directly relevant to human disease. Gene knockout of *Bbs6* and *Bbs8* in MEFs was confirmed via genotyping as described in Ross et al. (2005) and Tadenev et al. (2011) (Supplementary Figure S3H and I).

Filopodia are actin-based cell protrusions that sense the environment and are thus important for efficient cell migration (Amarachintha et al., 2015). Cells co-expressing mRFP-tagged Lifeact as a marker for the actin cytoskeleton and EGFP-tagged filopodia regulator Fascin-1 (EGFP-FSCN-1) were used for live cell imaging to visualize filopodia (Supplementary Videos). Fascin-1 bundles parallel F-actin filaments such as the ones found in filopodia. Thus, its function is restricted to filopodia, in contrast to other actin-dependent structures that contain anti-parallel filaments such as stress fibres (Vignjevic et al., 2006). This makes Fascin-1 a unique and reliable marker to study filopodia.

**Figure 1:**
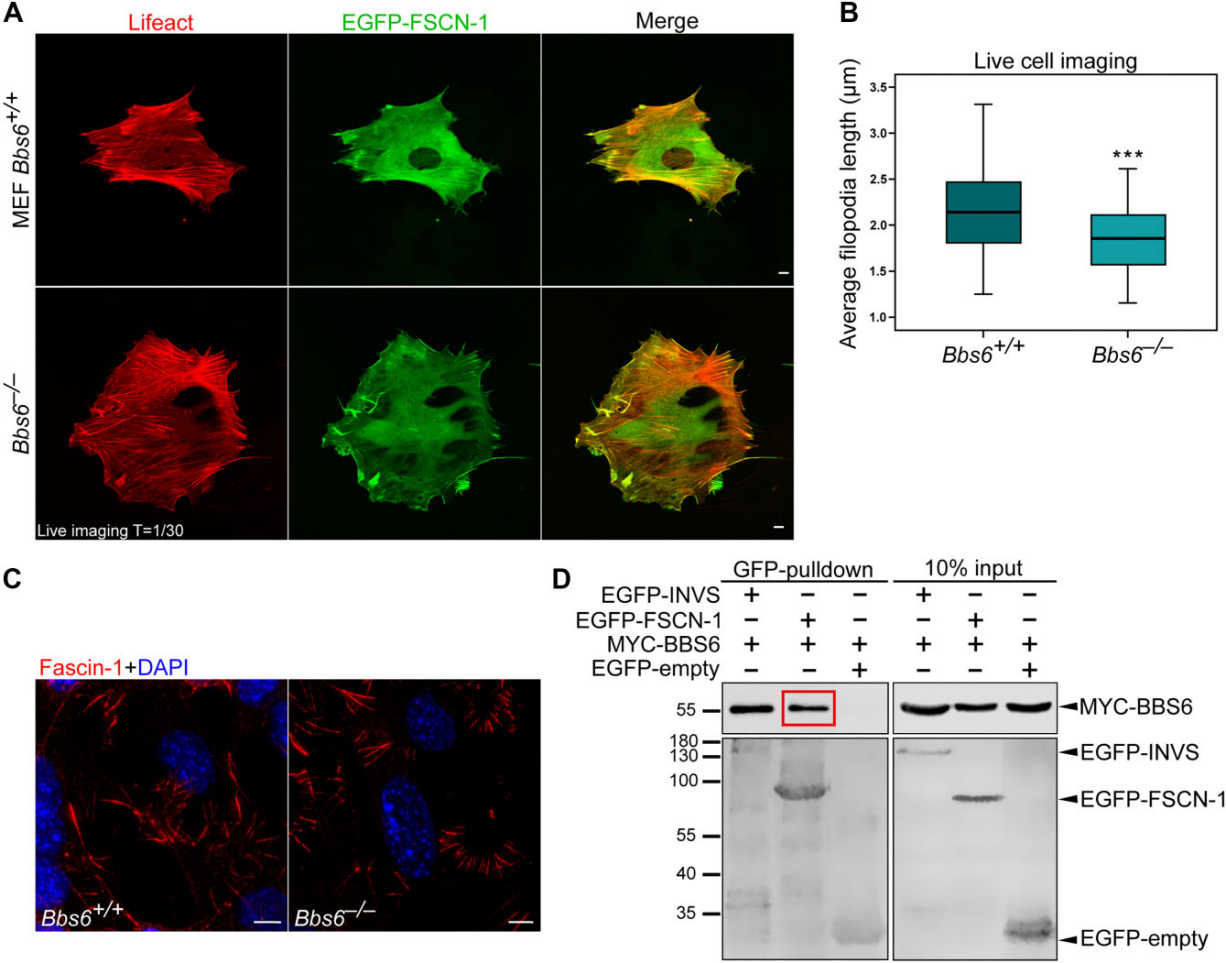
Loss of the ciliary protein BBS6 disrupts filopodia dynamics via the interaction with Fascin-1. (**A**) Live cell imaging of Lifeact (actin cytoskeleton) and EGFP-FSCN-1 (filopodia) in *Bbs6* wild-type (*Bbs6*^+/+^) and knockout (*Bbs6*^−/−^) MEFs at 48 h after transfection. Images represent 1 out of 30 timepoints in Supplementary Videos. Scale bar, 10 μm. (**B**) Average filopodia length by FiloQuant analysis shows significantly shorter filopodia upon *Bbs6* knockout. Mann–Whitney *U* test, ***P < 0.001. *n* (*Bbs6*^+/+^) = 93, *n* (*Bbs6*^−/−^) = 70. (**C**) *Bbs6*^+/+^ and *Bbs6*^−/−^ MEFs were fixed with methanol and stained for Fascin-1 (red). Fascin-1 localizes to filopodia in both *Bbs6*^+/+^ and *Bbs6*^−/−^ MEFs, showing no defect in localization upon *Bbs6* knockout. Scale bar, 10 μm. (**D**) Interaction study between Fascin-1 and BBS6. GFP-pulldown experiments were performed at 48 h after transfection as indicated in HEK293T cells. The interaction between EGFP-INVS and MYC-BBS6 was used as a positive control, and EGFP-empty served as a negative control. The red box shows the formation of a complex between EGFP-FSCN-1 and MYC-BBS6. The blots represent a cropped version of Supplementary Figure S1C. Experiments were repeated at least three times.

Analysis of the live cell imaging movies revealed that *Bbs6* knockout MEFs assembled shorter filopodia compared to wild-type cells (Figure 1A and B; Supplementary Videos S1 and S2), although the localizations of Fascin-1 to filopodia appeared to be similar in both fixed cells (Figure 1A and C), potentially due to the loss of filopodia stability during fixation. This filopodia phenotype is supported by previous studies, where their disruption was assumed to be the cause of migration defects observed in *Bbs6* knockout kidney medullary cells (Hernandez-Hernandez et al., 2013). Despite previous findings reported that loss of *Bbs8* hindered cell migration (Tobin et al., 2008; Hernandez-Hernandez et al., 2013), *Bbs8* knockout MEFs did not display a phenotype in filopodia length (Supplementary Figure S1A and B and Videos S3 and S4), suggesting that BBS8 is not involved in this aspect of environmental cell sensing.

Since Fascin-1 was the key actin-bundling protein organizing F-actin structures in filopodia (Kureishy et al., 2002; Vignjevic et al., 2006; Pfisterer et al., 2020), we adopted a candidate approach to determine whether the ciliary proteins BBS6 and BBS8 form a complex with Fascin-1. EGFP-FSCN-1, MYC-BBS6,

MYC-BBS8, and/or empty vector were co-expressed in HEK293T cells, and GFP-pulldown experiments were performed to assess complex formation. The previously described interaction between BBS6 and EGFP-tagged Inversin (EGFP-INVS) was used as a positive control (Patnaik et al., 2019). EGFP-FSCN-1 pulled down MYC-BBS6, but not MYC-BBS8 (Figure 1D; Supplementary Figure S1C). Taken together, these data indicate a regulation of filopodia by BBS6, but not BBS8, possibly via the association with Fascin-1.

### The functional interaction between BBS6 and Fascin-1 in cell migration is independent of transcriptional regulation

To understand whether BBS6 and Fascin-1 cooperate in the regulation of filopodia via influencing the expression of key regulatory proteins, we analyzed the expression levels of actin and ciliary components (Arl13b and polyglutamylated tubulin) upon loss of *Bbs6* or knockdown of *Fascin-1*.

**Figure 2:**
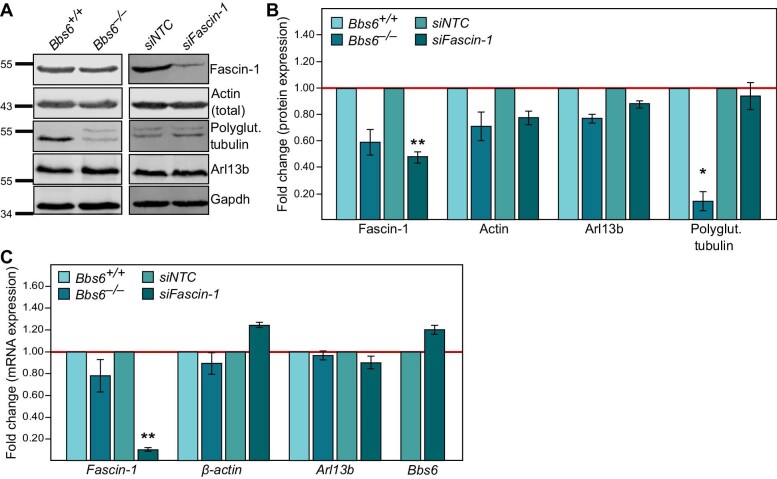
Transcription and translation of ciliary targets are not dependent on BBS6 or Fascin-1. (**A**) Protein levels of Fascin-1, actin, polyglutamylated tubulin (detected via Gt335), and Arl13b in MEFs determined by western blotting. (**B**) Quantitative analysis of the protein expression levels shown in **A** (normalized to Gapdh). The protein level of polyglutamylated tubulin was significantly downregulated in *Bbs6*^−/−^ MEFs (*P* = 0.02). The protein level of Fascin-1 was downregulated upon its knockdown. (**C**) mRNA levels of *Fascin-1, β-actin, Arl13b*, and *Bbs6* in MEFs measured by RT–qPCR. The mRNA level of *Fascin-1* was downregulated upon its knockdown. No significant differences in the mRNA levels were observed for all other genes. Student's *t*-test, *P < 0.05, **P < 0.01. Experimental procedures for both western blotting and RT–qPCR were repeated at least three times.

In *Bbs6* knockout cells, there was no change in the protein levels of Fascin-1, actin, and Arl13b, but the protein level of polyglutamylated tubulin was significantly reduced, indicating a regulatory role of BBS6 in the post-translational modification of tubulin (Figure 2A and B). The protein expression level of Fascin-1 appeared to be slightly, but not significantly, reduced upon *Bbs6* depletion. Furthermore, there was no change in the ubiquitination of Fascin-1 in *Bbs6*-depleted cells after treatment with the proteasome inhibitor MG132 (Supplementary Figure S3). Knockdown of *Fascin-1* in MEFs was performed using siRNA and validated by reverse transcription–quantitative polymerase chain reaction (RT–PCR), western blotting, and immunofluorescence (Supplementary Figure S3A–D). Upon knockdown of *Fascin-1*, there was no change in the expression levels of the target proteins, noting that the lack of a reliable antibody for BBS6 precluded us from analyzing the expression level of this protein. Loss of *Bbs6* did not affect the mRNA levels of *Fascin-1, β-actin*, and *Arl13b*, while knockdown of *Fascin-1* did not affect the mRNA levels of *β-actin, Arl13b*, and *Bbs6* (Figure 2C). Taken together, these results show that, except for polyglutamylated tubulin, the transcription and translation of these targets are not dependent on BBS6 or Fascin-1. These findings indicate that the functional crosstalk between BBS6 and Fascin-1 does not involve the control of protein expression or stability.

### Loss of *Fascin-1* is associated with a ciliary phenotype in MEFs

**Figure 3:**
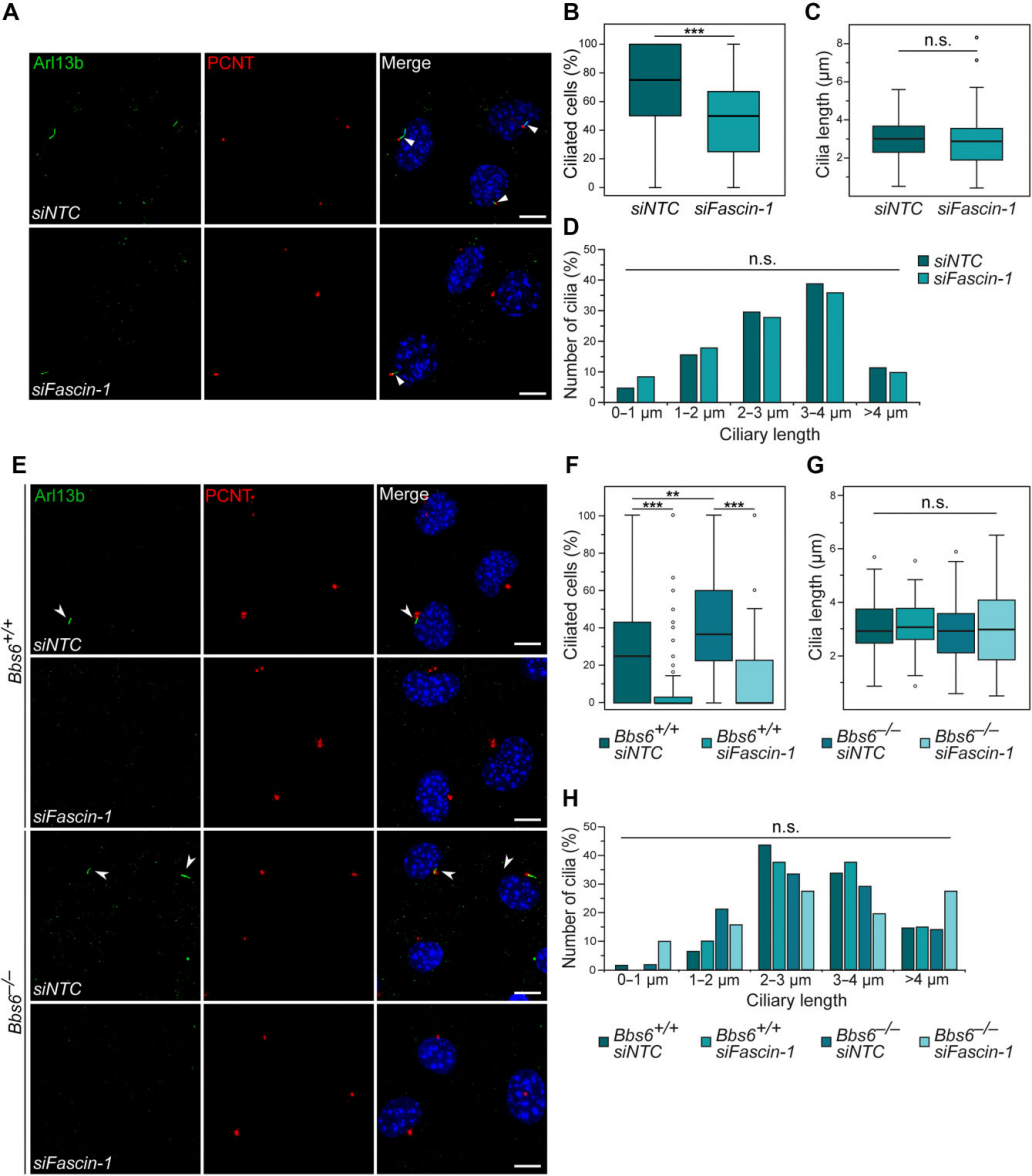
Loss of *Fascin-1* causes a ciliary phenotype. (**A**–**D**) Transfected MEFs were cultured in serum-depleted medium for 24 h to induce ciliation before fixation with 4% paraformaldehyde (PFA). (**A**) Primary cilia (arrow heads) were stained for the ciliary membrane marker Arl13b (green) and the basal body protein PCNT (red). Scale bar, 10 μm. (**B**) Quantification of ciliated cells upon knockdown of *Fascin-1*. Knockdown of *Fascin-1* significantly reduced the percentage of ciliated cells. *P* = 0.000037, *n* (*siNTC*) = 438, *n* (*siFascin-1*) = 395. (**C**) Analysis of cilia length upon knockdown of *Fascin-1. P* = 0.10461, *n* (*siNTC*) = 315, *n* (*siFascin-1*) = 212. (**D**) Ciliated cells were divided into five subclasses with cilia lengths of 0–1 μm, 1–2 μm, 2–3 μm, 3–4 μm, and >4 μm, respectively. Numbers of cilia were quantified in percentage. (**E**–**H**) *Bbs6*^+/+^ and *Bbs6*^−/−^ MEFs transfected with *siNTC* and *siFascin-1* were cultured in serum-depleted medium before fixation with 4% PFA. (**E**) Primary cilia (arrow heads) were stained for Arl13b (green) and PCNT (red). Scale bar, 10 μm. (**F**) Quantification of ciliated cells. *n* (*Bbs6*^+/+^, *siNTC*) = 462, *n* (*Bbs6*^+/+^, *siFascin-1*) = 458, *n* (*Bbs6*^−/−^, *siNTC*) = 393, *n* (*Bbs6*^−/−^, *siFascin-1*) = 363. (**G**) Analysis of cilia length. *n* (*Bbs6*^+/+^, *siNTC*) = 123, *n* (*Bbs6*^+/+^, *siFascin-1*) = 42, *n* (*Bbs6*^−/−^, *siNTC*) = 156, *n* (*Bbs6*^−/−^, *siFascin-1*) = 51. (**H**) Ciliated cells were divided into five subclasses of different cilia lengths, and numbers of cilia were quantified in percentage. Mann–Whitney *U* test was performed in **B**–**D** and **F**–**H**. n.s. indicates *P* > 0.05, **P < 0.01, ***P < 0.001. The data represent at least three independent experiments.

The interaction between Fascin-1 and BBS6 and the filopodia phenotype in *Bbs6*-depleted cells suggest that these proteins could cooperate in other similar cellular processes. Although the primary cilium is predominantly a microtubule-based structure, actin-related proteins also play an important role in ciliogenesis (Bershteyn et al., 2010; Kim et al., 2010, 2015; Pitaval et al., 2010; Liang et al., 2016). To investigate whether this is true for Fascin-1, we quantified the number and length of primary cilia after *Fascin-1* knockdown. Transfection with *siFascin-1* in MEFs led to a significantly lower percentage of ciliated cells (∼50%) compared to transfection with non-targeting control siRNA (*siNTC*, ∼80%) (Figure 3A and B). Two independent siRNAs targeting the *Fascin-1* gene likewise generated same defects in ciliogenesis (Supplementary Figure S3F). Almost no changes in cilia length were observed following *Fascin-1* knockdown (Figure 3C and D), suggesting that the ciliation defect is most likely a consequence of failed initiation.

Loss of *Bbs6* has been shown to increase ciliation in kidney medullary cells (Volz et al., 2021). To further analyze the interplay between BBS6 and Fascin-1 in ciliogenesis, we analyzed whether knockdown of *Fascin-1* in *Bbs6-*depleted cells could rescue the ciliary phenotype. It is noted that *Bbs6* wild-type MEFs transfected with *siNTC* contained ∼30% ciliated cells (Figure 3F), much less compared with the siNTC-transfected MEFs in Figure 3B, which could be caused by different genetic backgrounds of these immortalized cell lines and the different methods used for immortalization. Similarly, knockdown of *Fascin-1* in *Bbs6* wild-type cells reduced the percentage of ciliated cells (Figure 3E and F). As expected, *Bbs6*-depleted MEFs had a significantly higher percentage of ciliated cells, which was attenuated by *Fascin-1* knockdown (Figure 3E and F), while cilia lengths were unaltered (Figure 3G and H). These results show that Fascin-1 is required for ciliogenesis in MEFs and potentially acts antagonistically to BBS6 as a positive regulator of ciliogenesis.

### Fascin-1 localizes transiently to primary cilia

**Figure 4:**
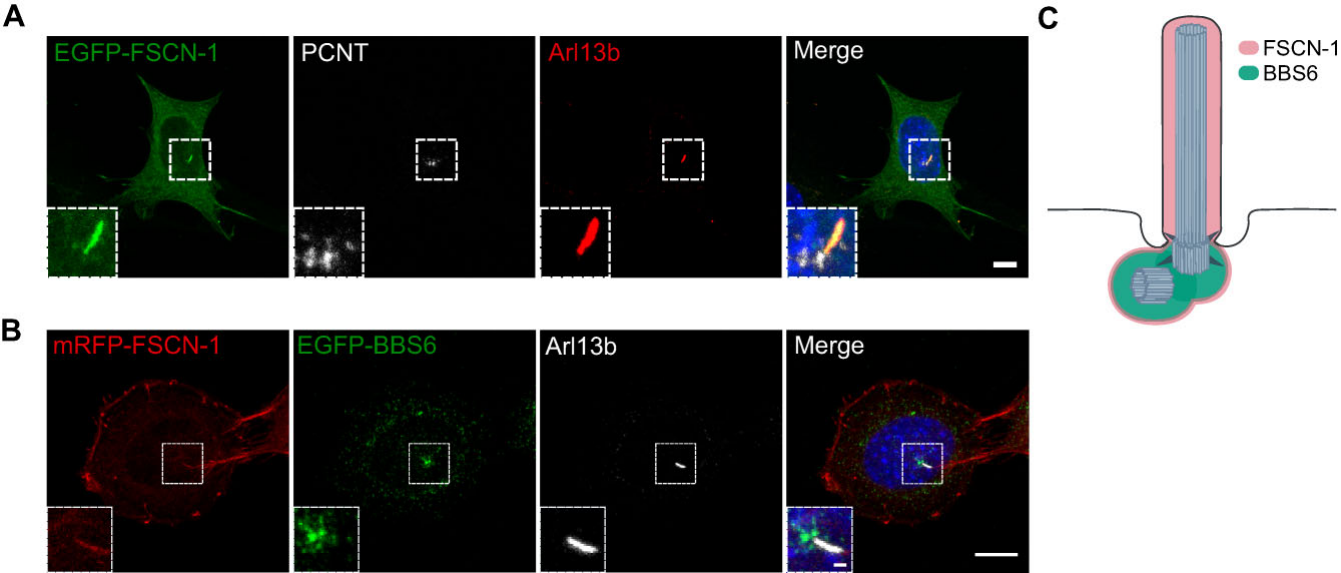
Fascin-1 localizes transiently to primary cilia. (**A**) EGFP-FSCN-1-overexpressing MEFs were cultured in serum-depleted medium for 24 h to induce ciliation and co-stained for PCNT and Arl13b. Scale bar, 8 μm. (**B**) MEFs co-expressing mRFP-FSCN-1 and EGFP-BBS6 were cultured in serum-depleted medium for 24 h and stained for Arl13b. mRFP-FSCN-1 localized to the ciliary axoneme, whereas EGFP-BBS6 stayed at and around the basal body. Scale bar, 10 μm and 1 μm (inset). (**C**) Graphical representation of the localization of BBS6 and Fascin-1 at the primary cilium. Fascin-1 localizes both at the basal body and along the ciliary axoneme, whereas BBS6 translocates only to the basal body.

Fascin-1 has been shown to localize to primary cilia of murine fibroblasts, where it was suggested to assist actin-based ciliary decapitation (Phua et al., 2017). To verify this Fascin-1 localization, we overexpressed EGFP-FSCN-1 in MEFs and performed fluorescence microscopy. A distinct localization of EGFP-FSCN-1 both at the basal body and along the ciliary axoneme was observed in a small proportion of cells (Figure 4A), suggesting that this recruitment is either transient or linked to a specific state of ciliogenesis. We were not able to see endogenous Fascin-1 localizing to primary cilia, which might be due to technical limitations of microscopy resolution.

The actin- and microtubule-binding properties of Fascin-1 can be influenced by manipulating two phosphorylation sites, S39 and S274, within the two actin-binding domains (Villari et al., 2015). We therefore tested whether the phospho-mimetic construct with decreased actin-binding capacity (S274D) showed stronger localization to primary cilia, as it binds to microtubules more efficiently. However, neither this mutant nor other mutant forms (S39A, S39D, and S274A that are known to influence cytoskeletal binding) of Fascin-1 could increase its ciliary localization (Supplementary Figure S4).

Since Fascin-1 was identified in a complex with BBS6, we wondered whether both proteins colocalized at the primary cilium. Co-expression of both mRFP-tagged Fascin-1 (mRFP-FSCN-1) and EGFP-BBS6 showed a distinct localization of Fascin-1 inside the ciliary axoneme, supporting our findings with EGFP-FSCN-1 (Figure 4B). EGFP-BBS6 localized at and around the basal body, which is in line with previous studies (Kim et al., 2005). It is important to note that BBS6 has never been shown to enter the ciliary axoneme. Although the signal of Fascin-1 localized at the basal body was not as strong as that within the axoneme (Figure 4A), we could still suggest that Fascin-1 and BBS6 do colocalize at the basal body (Figure 4C).

Taken together, we identified that Fascin-1 is transiently recruited to the cilium, potentially colocalizing with BBS6 around the basal body.

### Fascin-1 interacts with the PCP regulator Inversin

**Figure 5:**
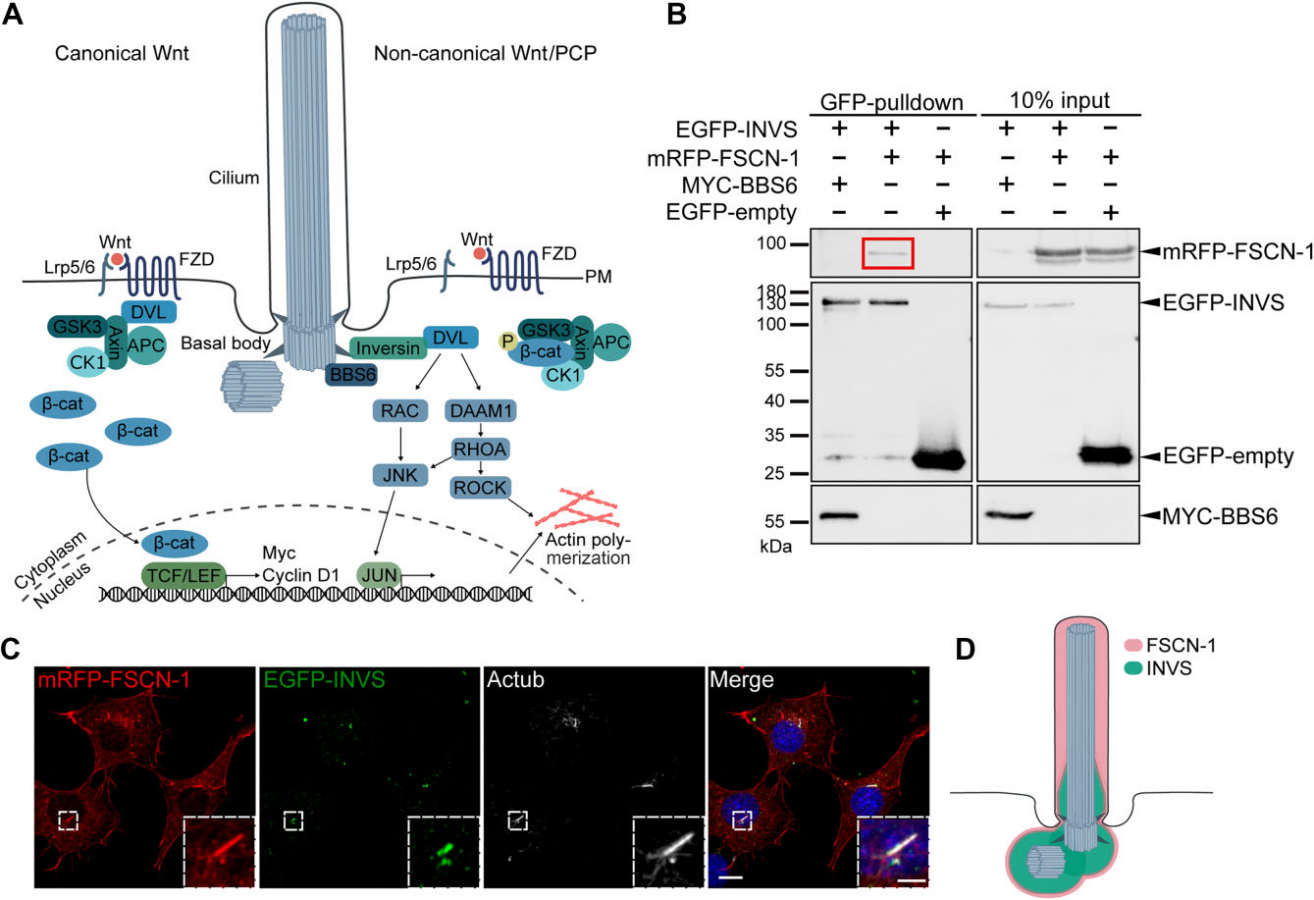
Fascin-1 interacts and colocalizes with the PCP regulator Inversin at primary cilia. (**A**) Graphical representation of the canonical Wnt and non-canonical Wnt/PCP signalling pathways. Upon activation of canonical Wnt signalling via the Wnt ligand binding to a coreceptor complex consisting of Lrp5/6 and Frizzled, Dishevelled inactivates the β-catenin degradation complex (Axin, GSK3, APC, and CK1). β-catenin accumulates and enters the nucleus, where it acts as the coactivator of transcription factor TCF/LEF that activates the transcription of Wnt target genes such as Cyclin D1 and Myc. Upon activation of non-canonical Wnt/PCP signalling, Dishevelled is translocated by Inversin, so that β-catenin gets degraded. Dishevelled activates downstream signalling cascades via regulating Rac, Daam1, and Rho GTPases, consequently activating actin networks. β-cat, β-catenin; DVL, Dishevelled; FZD, Frizzled; PM, plasma membrane. (**B**) Interaction study between Fascin-1 and Inversin. GFP-pulldown experiments were performed at 48 h after transfection as indicated in HEK293T cells. The interaction between EGFP-INVS and MYC-BBS6 was used as a positive control, and EGFP-empty served as a negative control. The red box shows the formation of a complex between mRFP-FSCN-1 and EGFP-INVS. Experiments were repeated at least three times. (**C**) MEFs co-expressing mRFP-FSCN-1 and EGFP-INVS were cultured in serum-depleted medium for 24 h to induce ciliation and stained for the ciliary marker protein acetylated tubulin (Actub). mRFP-FSCN-1 localized along the ciliary axoneme. EGFP-INVS localized to the basal body and lower part of the axoneme (presumably the transition zone). Scale bar, 10 μm and 3 μm (inset). (**D**) Graphical representation of the ciliary localization of mRFP-FSCN-1 and EGFP-INVS in MEFs. Fascin-1 localizes to the complete ciliary axoneme, whereas Inversin localizes at the basal body and the transition zone, thus showing a colocalization of two proteins at the transition zone.

Since loss of *Fascin-1* results in a reduction in ciliated cells, we sought to further determine the downstream effects. Primary cilia regulate several signalling pathways, including Wnt signalling, which can modulate actin network assembly (Figure 5A; Gerdes et al., 2007; Corbit et al., 2008; May-Simera et al., 2010; Cui et al., 2013; McMurray et al., 2013; Balmer et al., 2015; May-Simera et al., 2015; Wang et al., 2017). BBS6 and Inversin interact and regulate the switch from canonical to non-canonical Wnt signalling (Simons et al., 2005; Gerdes et al., 2007; Patnaik et al., 2019). Since Fascin-1 was identified in a complex with BBS6, we hypothesized that it might also interact with Inversin. We overexpressed mRFP-FSCN-1, EGFP-INVS, and/or empty vector and then performed GFP-pulldown experiments to assess complex formation. The interaction between MYC-BBS6 and EGFP-INVS was used as a positive control (Patnaik et al., 2019). EGFP-INVS pulled down mRFP-FSCN-1, indicating an interaction between both proteins (Figure 5B).

To analyze whether Fascin-1 and Inversin interact at the primary cilium, we performed fluorescence microscopy of EGFP-INVS and mRFP-FSCN-1 in ciliated cells (Figure 5C). EGFP-INVS localized to the basal body and lower part of the axoneme, presumably the transition zone, representing a well-characterized localization for Inversin (Shiba et al., 2009; Veland et al., 2013). Since mRFP-FSCN-1 localized along the complete ciliary axoneme, we suggest that Fascin-1 and Inversin might colocalize and interact at the transition zone and around the basal body (Figure 5D).

### Fascin-1 is involved in the regulation of ciliary Wnt signalling

**Figure 6:**
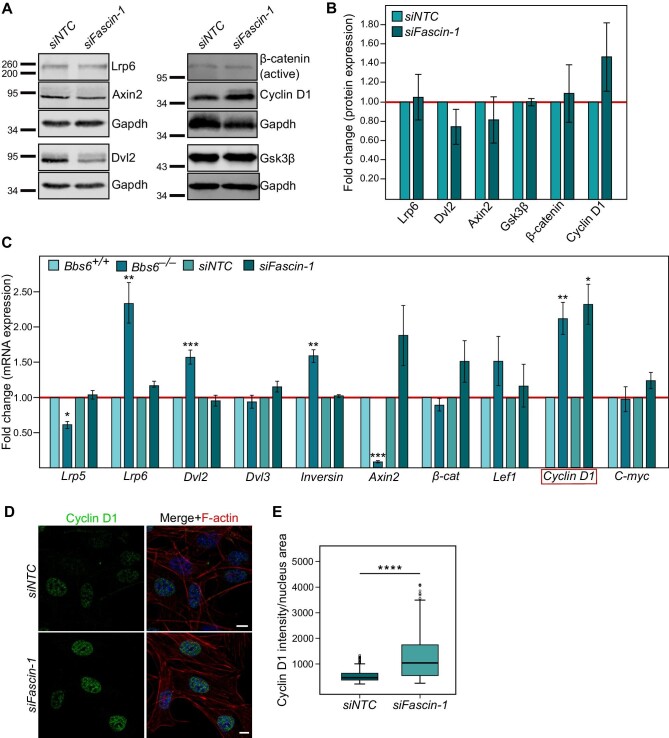
Fascin-1 transcriptionally regulates downstream ciliary Wnt signalling. (**A**) Protein levels of key Wnt pathway components Lrp6, Axin2, Dvl2, active β-catenin, Cyclin D1, and Gsk3β in *Fascin-1* knockdown MEFs determined by western blotting (Gapdh serving as respective control). (**B**) Quantitative analysis of the protein expression levels shown in **A** (normalized to Gapdh). No significant differences were detected, although Cyclin D1 level trended to be increased upon *Fascin-1* knockdown. Student's *t*-test, *P* > 0.05. (**C**) mRNA levels of Wnt signalling genes upon *Fascin-1* knockdown and/or *Bbs6* knockout in MEFs measured by RT–qPCR. Data are shown as fold changes in comparison to the respective wild-type or *siNTC* control (red line). Student's *t*-test, *P < 0.05, **P < 0.01, ***P < 0.001. (**D**) Visualization of Cyclin D1 (green) inside the nucleus of MEFs transfected with *siNTC* or *siFascin-1* at 48 h prior to fixation with 4% PFA. Scale bar, 10 μm. (**E**) Quantification of the fluorescence intensity of Cyclin D1 inside the nucleus of MEFs measured with Fiji in relation to the nucleus area. Mann–Whitney *U* test, *P* = 8.98 × 10^−26^, *n* (*siNTC*) = 297, *n* (*siFascin-1*) = 282. The data represent at least three independent experiments.

The interaction between Fascin-1 and the PCP regulator Inversin suggested a function for Fascin-1 in cilia-related non-canonical Wnt/PCP signalling. To test this, we examined the levels of key Wnt signalling proteins after knockdown of *Fascin-1.* We performed western blotting with antibodies against a variety of Wnt proteins, such as Lrp6, Axin2, Dvl2, active β-catenin, Cyclin D1, and Gsk3β (Figure 5A). Although none of the proteins showed significant changes in their expression levels upon loss of *Fascin-1*, we observed an upregulation trend of Cyclin D1, a downstream target of canonical Wnt (Figure 6A and B). We also noted a downregulation trend of Dvl2, a regulator of non-canonical Wnt/PCP.

Accordantly, knockdown of *Fascin-1* in MEFs significantly increased the mRNA expression level of *Cyclin D1* but not other Wnt effectors (Figure 6C). Loss of *Bbs6* significantly enhanced *Cyclin D1* mRNA level and also significantly changed the mRNA levels of other Wnt pathway components, such as *Lrp5/6, Dvl2, Inversin*, and *Axin2* (Figure 6C). Since loss of *Bbs6* activates canonical Wnt signalling (Gerdes et al., 2007; Patnaik et al., 2019; Volz et al., 2021), this might be facilitated via a transcriptional upregulation of *Cyclin D1* and *Lrp6* and downregulation of *Axin2*. Dvl2 is known to interact with Inversin in mediating non-canonical Wnt/PCP. Thus, the transcriptional upregulation of *Dvl2* and *Inversin* might be a mechanism to compensate for the hyperactivation of canonical Wnt due to loss of *Bbs6*.

The upregulation of Cyclin D1 protein level upon loss of *Fascin-1* was further confirmed by immunocytochemistry. The intensity of Cyclin D1 inside the nucleus was significantly enhanced upon knockdown of *Fascin-1* (Figure 6D and E), suggesting an activation of canonical Wnt signalling (Shtutman et al., 1999; Tetsu and McCormick, 1999). Since primary cilia were shown to modulate Wnt signalling (Gerdes et al., 2007; Corbit et al., 2008), these data indicate the upregulation of canonical Wnt signalling being a consequence of the ciliary phenotype upon loss of *Fascin-1*.

### Fascin-1 cooperates with BBS6 in modulating Wnt signalling

**Figure 7:**
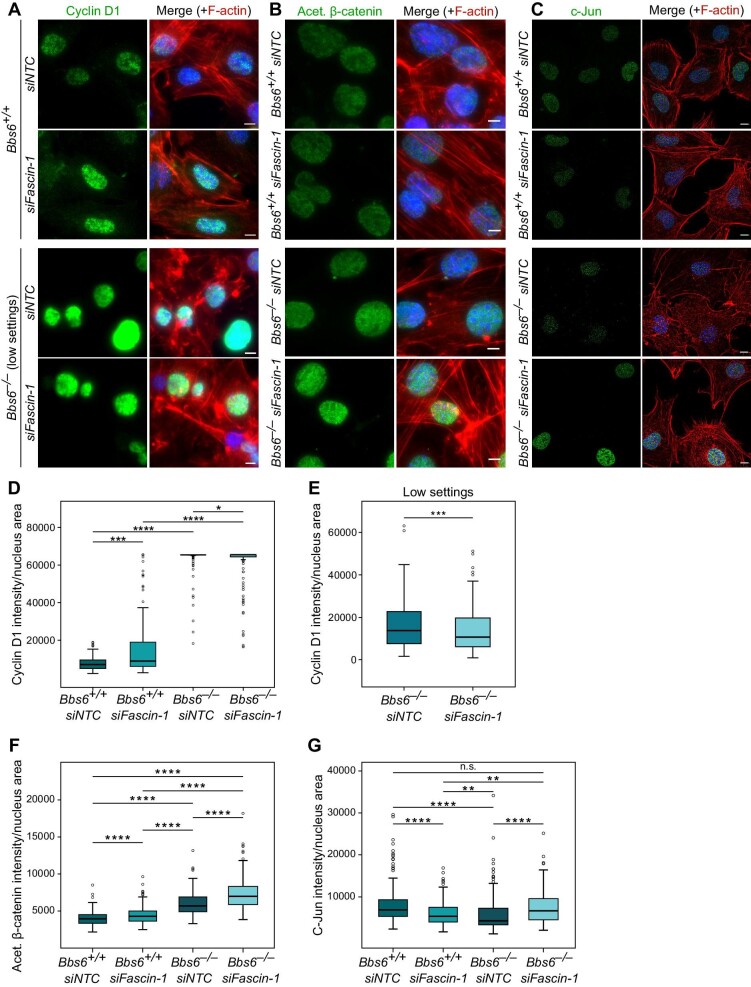
Fascin-1 regulates ciliary Wnt signalling in cooperation with BBS6. (**A**–**C**) Visualization of Cyclin D1, acetylated β-catenin, and c-Jun inside the nucleus of *Bbs6*^+/+^ and *Bbs6*^−/−^ MEFs transfected with *siNTC* or *siFascin-1* at 48 h prior to fixation with 4% PFA. For Cyclin D1 in *Bbs6*^−/−^ MEFs (**A**, lower panel), microscope illumination settings were reduced to avoid saturation and enable subsequent analysis. Scale bar, 10 μm. (**D**) Quantification of the fluorescence intensity of Cyclin D1 inside the nucleus in relation to the nucleus area. *n* (*Bbs6*^+/+^, *siNTC*) = 157, *n* (*Bbs6*^+/+^, *siFascin-1*) = 132, *n* (*Bbs6*^−/−^, *siNTC*) = 157, *n* (*Bbs6*^−/−^, *siFascin-1*) = 157. (**E**) Quantification of the fluorescence intensity of Cyclin D1 inside the nucleus under lower microscopy illumination settings. *P* = 0.002, *n* (*Bbs6*^−/−^, *siNTC*) = 157, *n* (*Bbs6*^−/−^, *siFascin-1*) = 128. (**F**) Quantification of the fluorescence intensity of acetylated β-catenin inside the nucleus. *n* (*Bbs6*^+/+^, *siNTC*) = 282, *n* (*Bbs6*^+/+^, *siFascin-1*) = 211, *n* (*Bbs6*^−/−^, *siNTC*) = 309, *n* (*Bbs6*^−/−^, *siFascin-1*) = 327. (**G**) Quantification of the fluorescence intensity of c-Jun inside the nucleus. *n* (*Bbs6*^+/+^, *siNTC*) = 166, *n* (*Bbs6*^+/+^, *siFascin-1*) = 98, *n* (*Bbs6*^−/−^, *siNTC*) = 215, *n* (*Bbs6*^−/−^, *siFascin-1*) = 75. Mann–Whitney *U* test was performed in **D**–**G**. n.s. indicates *P* > 0.05, *P < 0.05, **P < 0.01, ***P < 0.001, ****P < 0.0001. The data represent at least three independent experiments.

Given that loss of *Bbs6* and loss of *Fascin-1* both led to increased *Cyclin D1* mRNA levels, we next tested whether BBS6 and Fascin-1 acted synergistically in this pathway. We quantified nuclear Cyclin D1 levels in *Bbs6* wild-type and knockout MEFs with or without *Fascin-1* knockdown (Figure 7A, D, and E). In *Bbs6* wild-type cells, knockdown of *Fascin-1* led to an increase in nuclear Cyclin D1 level (Figure 7A and D; Volz et al., 2021). Since the fluoresence intensity of Cyclin D1 in *Bbs6* knockout cells was too high, microscope illumination settings had to be reduced to avoid saturation and enable subsequent analysis (Figure 7A, lower panel). Interestingly, we observed a significant decrease in nuclear Cyclin D1 level in *Bbs6* knockout cells upon *Fascin-1* knockdown (Figure 7A and E).

Besides Wnt signalling, the cell cycle could also control the nuclear localization of Cyclin D1. Previous research has demonstrated that the absence of BBS6 does not significantly affect the G1/S phase transition that regulates the translocation of Cyclin D1 (Kim et al., 2005; Patnaik et al., 2019). To preclude the possibility that Fascin-1 interferes with the G1/S phase transition to affect the nuclear localization of Cyclin D1, we performed proliferation assays (Supplementary Figure S3E). We found that *Fascin-1* knockdown in MEFs did not affect cell proliferation, leading us to conclude that the upregulation of nuclear Cyclin D1 levels is a result of enhanced canonical Wnt signalling rather than the disturbance of the cell cycle.

We further examined two other Wnt signalling targets, acetylated β-catenin and c-Jun, to determine whether their changes upon *Fascin-1* knockdown and/or *Bbs6* knockout were correlated with changes in Cyclin D1 levels. β-catenin, one of the canonical Wnt effectors, is acetylated at Lys49 via CREB-binding protein, regulating its transcriptional activity in a promoter-specific fashion (Wolf et al., 2002). In wild-type MEFs, acetylated β-catenin was restricted to the nucleus (Figure 7B), making it a suitable readout for Wnt activity. Significantly enhanced nuclear levels of acetylated β-catenin were seen in either *Fascin-1* knockdown or *Bbs6* knockout cells (Figure 7B and F). However, knockdown of *Fascin-1 in Bbs6*-depleted cells did not reverse this phenotype (Figure 7B and F). The different expression patterns of Cyclin D1 and acetylated β-catenin suggest that BBS6 and Fascin-1 do not completely overlap in terms of their functional effects on canonical Wnt signalling. c-Jun is a downstream target of non-canonical Wnt/PCP. Either knockdown of *Fascin-1* or knockout of *Bbs6* led to a decrease in the nuclear level of c-Jun (Figure 7C and G). Moreover, knockdown of *Fascin-1* in *Bbs6*-depleted cells restored the levels of c-Jun inside the nucleus. Taken together, loss of *Fascin-1* is associated with the upregulation of two canonical Wnt targets, Cyclin D1 and acetylated β-catenin, and the downregulation of a non-canonical Wnt/PCP target, c-Jun.

## Discussion

Previous studies have shown that several ciliary proteins are not only associated with ciliary function but also with actin regulation and subsequent cell migration (Yin et al., 2009; Kim et al., 2010; May-Simera et al., 2016). In particular, loss of *Bbs6* enhances actin stress fibres and focal adhesions, two key components of the actin network, in kidney medullary cells (Hernandez-Hernandez et al., 2013). Filopodia represent another key element of the actin cytoskeleton, as they extend beyond the leading edge of lamellipodia to sense the environment and are thus important to induce cell migration. In this study, we found that filopodia in *Bbs6* knockout MEFs were significantly shorter in comparison to those in wild-type cells, suggesting a defect in environmental sensing. Furthermore, we identified BBS6 in a complex with the actin regulator Fascin-1. As a functional downstream target of Rho signalling (Jayo et al., 2012), Fascin-1 bundles parallel actin filaments, stabilizing key migratory structures such as filopodia (Kureishy et al., 2002; Vignjevic et al., 2006; Pfisterer et al., 2020). Although BBS8 was previously reported to be associated with focal adhesions and stress fibres similarly to BBS6 (Hernandez-Hernandez et al., 2013), we did not see a defect in filopodia length upon loss of *Bbs8* or find BBS8 forming a complex with Fascin-1. Interestingly, the expression and localization of Fascin-1 were not altered in *Bbs6*-depleted cells. Thus, we conclude that, although Fascin-1 and BBS6 interact with each other, the filopodia defect in *Bbs6*-depleted cells is not a result of its direct regulation of Fascin-1. Since BBS6 was shown to be involved in the regulation of ciliary Wnt signalling, which affects the downstream actin networks involving Rho signalling, we suggest that the filopodia defect in *Bbs6*-deficient cells might be an indirect effect of Wnt signalling, which will be discussed later in more detail.

BBS6 was previously shown to be translocated into the nucleus, where it is transcriptionally active through interacting with chromatin remodelling factors (Scott et al., 2017). Overexpression of *bbs6* in zebrafish leads to differentially regulated gene levels of *Fascin-*1 and several actin regulators, as shown by RNA sequencing (Scott et al., 2017). Thus, it is highly likely that loss of *Bbs6* affects the transcription of actin regulators, further affecting downstream filopodia networks. However, it has to be noted that the mRNA level of *Fascin-1* itself was not altered upon loss of *Bbs6* in our study.

Our data further indicate a positive role for Fascin-1 in ciliogenesis. Although primary cilia are predominantly microtubule-based structures, actin-related proteins have long been found to affect ciliogenesis. During the initial stages of ciliogenesis, many actin regulators, such as Arp2/3, focal adhesion kinase, vinculin, paxillin, and Rho GTPases, are involved in the maturation of the mother centriole and positioning of the basal body (Brücker et al., 2020). In cycling cells, polymerized F-actin is associated with decreased ciliogenesis (Bershteyn et al., 2010; Kim et al., 2010, 2015; Liang et al., 2016). In this study, Fascin-1 was identified to join the long list of actin-binding proteins in regulating ciliogenesis. Loss of *Fascin-1* reduced the number of ciliated cells without affecting cilia length, suggesting a role for Fascin-1 in the initiation of ciliogenesis. It is plausible that Fascin-1 is involved in the initiation of ciliogenesis by maintaining a stable actin network by which ciliary vesicles can be transported (Hong et al., 2015; May-Simera et al., 2016; Wu et al., 2018). In support of these data, Fascin-1 was found to interact with Nespin-2, an actin scaffold at the outer nuclear lamina (Jayo et al., 2016). Nesprin-2 is required for precise trafficking of Arp2-dependent preciliary vesicles during centriole maturation, suggesting that the role of Fascin-1 during early ciliogenesis might be facilitated via its interaction with Nesprin-2 (Fan et al., 2020). Interestingly, an affinity proteomics screen provided the first hints that BBS proteins might also interact with proteins of the Nesprin family (Boldt et al., 2016).

We also found that Fascin-1 acted antagonistically to BBS6 on ciliogenesis. BBS6 is a *bona fide* ciliary protein as part of a chaperonin-like complex essential for the initial assembly of the BBSome (Seo et al., 2010), a multiprotein complex required for ciliary trafficking (Nachury et al., 2007; Wei et al., 2012). Thus, depletion of *Bbs6* correlates with cell type-specific ciliary phenotypes, e.g. reduced cilia number and length in inner medullary collecting duct cells, as well as retinal pigment epithelium cell lines and tissues, whereas enhanced cilia number and length in kidney medullary cells (Scott et al., 2017; Patnaik et al., 2019; Volz et al., 2021). In this study, we showed that loss of *Bbs6* in MEFs enhanced cilia number, although the length was not affected. Mechanistically, this might be due to its interaction with MACF1, an actin- and microtubule-binding protein involved in the docking of preciliary vesicles in the initial steps of ciliogenesis (May-Simera et al., 2016). Similarly, the interaction between BBS6 and Fascin-1 might also be important for preciliary vesicle docking during early ciliogenesis, a process that requires both stable actin and microtubule networks. In support of this hypothesis, we showed that overexpressed BBS6 and Fascin-1 colocalized at the ciliary base. It is likely that Fascin-1 is required for the function of BBS6 in chaperoning other important ciliary trafficking proteins. It is still unclear whether Fascin-1 is necessary for the function of BBS6 in ciliogenesis, or whether, the other way round, BBS6 is required for the function of Fascin-1 in ciliogenesis. Since the ciliary phenotypes observed upon loss of *Fascin-1* and loss of *Bbs6* are not concordant, the balance between two proteins might be crucial to regulate ciliogenesis in a fine-tuned mechanism.

Since actin proteins are highly involved in many steps of ciliogenesis, it is not surprising that F-actin itself and many actin regulators have recently been identified inside primary cilia (Nager et al., 2017; Phua et al., 2017; Kiesel et al., 2020). In this work, we showed that, albeit transiently, overexpressed Fascin-1 also localizes to primary cilia, consistent with the finding in a previous study (Phua et al., 2017). Fascin-1 may aid in the process of ectocytosis at the ciliary tip, which is one of the functions of F-actin in cilia and serves as a means of cilia disassembly (Phua et al., 2017; Kiesel et al., 2020). However, we saw Fascin-1 localizing along the complete axoneme but not accumulating at the ciliary tip. Besides regulating F-actin bundles, Fascin-1 is also able to bind to and regulate microtubules to control focal adhesion dynamics and the speed of cell migration independently of its actin-binding function (Villari et al., 2015). This raises the possibility that the localization of Fascin-1 inside primary cilia might be co-dependent on both F-actin and microtubule regulation, although we did not find an increase in ciliary Fascin-1 levels upon cytoskeletal disruption. Taken together, we identified Fascin-1 as a possible ciliary protein, localizing transiently to primary cilia and contributing to cilia assembly. These data suggest that mutations in Fascin-1 might be associated with ciliopathies.

Interestingly, the second isoform of Fascin, retinal Fascin-2, is highly homologous to Fascin-1 and has many characteristics that are reminiscent of other ciliopathy proteins. Fascin-2 localizes to the inner and outer segments of photoreceptor cells (the outer segment being a highly specialized primary cilium) and actin-based stereocilia of the cochlea (Yokokura et al., 2005; Lin-Jones and Burnside, 2007; Perrin et al., 2013). Mutations in the *Fascin-2* gene are associated with retinopathies and progressive hearing loss due to shortened stereocilia bundles in mice (Yokokura et al., 2005; Perrin et al., 2013; Liu et al., 2018). There is also evidence that patient mutations in *Fascin-2* lead to macular degeneration and cone dystrophy, both of which are common ciliopathy phenotypes (Wada et al., 2003; Gui et al., 2018). These data raise the possibility that Fascin-2 and possibly its isoform Fascin-1, the focus of this study, are *bona fide* ciliopathy proteins. Interestingly, overexpression of *bbs6* in zebrafish leads to differential expression of *Fascin-2* (Scott et al., 2017). Since there is still a certain percentage of ciliopathy patients with undiagnosed mutations, Fascin-1 and Fascin-2 might be interesting candidates to screen in ciliopathy patients.

It is necessary to consider the downstream function of Fascin-1 in relation to ciliary dysfunction and actin dynamics. The non-canonical Wnt/PCP signalling pathway bridges ciliogenesis and actin networks. Upon activation of the non-canonical Wnt/PCP pathway, Frizzled receptor activation recruits Dishevelled to the plasma membrane via Inversin, where it activates formins such as Daam1 and Rho GTPases that consequently regulate downstream actin networks. BBS6 interacts with Inversin and facilitates its transport to the base of the cilium, activating non-canonical Wnt/PCP signalling (Patnaik et al., 2019). Therefore, BBS6 is reported to be a positive regulator of non-canonical Wnt/PCP signalling (Gerdes et al., 2007; May-Simera et al., 2018; Patnaik et al., 2019; Volz et al., 2021). In this study, we showed that both BBS6 and Fascin-1 positively regulated a downstream non-canonical Wnt/PCP target, c-Jun. We also showed that Fascin-1 interacted and colocalized with the PCP effector Inversin at primary cilia. These data indicate that Fascin-1 acts as a novel effector for non-canonical Wnt/PCP signalling. Taken together, we suppose that Fascin-1 acts in a complex with both BBS6 and Inversin in regulating non-canonical Wnt/PCP signalling.

Also, the role of Fascin-1 in cancer cells, where canonical Wnt signalling is often enhanced, has to be considered (Shang et al., 2017). In several cancer types, Fascin-1 seems to be a positive regulator of Wnt signalling, in contrast to our data. Reducing the expression level of Fascin-1 in breast cancer cells led to a decrease in the expression levels of β-catenin and Cyclin D1, which in turn impacted the growth of tumour cells (Barnawi et al., 2020). In the same type of cells, Fascin-1 was shown to interact with the non-canonical Wnt/PCP downstream target Daam1, promoting cancer cell migration (Hao et al., 2021). In human colorectal cancer, five putative TCF-binding sites in the untranslated region of the *Fascin-1* promoter were identified, and the *Fascin-1* gene was transactivated via TCF/LEF transcription factors that drive canonical Wnt signalling (Vignjevic et al., 2007). However, it has to be noted that the expression of Fascin-1 is naturally enhanced in cancer cell lines (Jayo and Parsons, 2010). Furthermore, loss of *Fascin-1* in melanoblasts was associated with less Cyclin D1-positive nuclei (Ma et al., 2013). Combining previous reports with our finding that Fascin-1 is a negative regulator of canonical Wnt signalling in MEFs, we conclude that the role of Fascin-1 in Wnt signalling is highly cell type-specific.

There is also evidence for Fascin-1 in the regulation of Wnt signalling via cytonemes, specialized filopodia, that act as signalling hubs for the Wnt pathway in zebrafish and non-cancerous cell lines (Routledge and Scholpp, 2019). Downstream non-canonical Wnt/PCP signalling controls the emergence of cytonemes, which can then transport Wnt molecules to recipient cells, inducing canonical Wnt cascades (Stanganello et al., 2015; Mattes et al., 2018; Rosenbauer et al., 2020). Moreover, cytonemes can also distinguish between different types of signals and selectively grow in the direction of a preferred Wnt signal (Junyent et al., 2020). Hence, cytonemes are important for distributing and receiving Wnt signals. Because Fascin-1 is required for cytoneme formation (Mattes and Scholpp, 2018; Junyent et al., 2020), these data depict an involvement of Fascin-1 in Wnt signalling of non-cancerous cells via the regulation of cytonemes. Our study showed a defect in filopodia length upon loss of *Bbs6*, suggesting that BBS6 may be also involved in the sensing function of cytonemes and the concomitant distribution of Wnt signals from or to other cells.

Taken together, cilia, Wnt signalling, and actin networks are tightly interconnected and affect each other in a complicated feedback mechanism, making it hard to define the explicit regulations of ciliary proteins, Fascin-1, and Wnt. More research will help to understand the complex interplay among ciliogenesis, Wnt signalling, and actin regulation, shedding light on how ciliopathies affect cellular homeostasis.

## Materials and methods

### Cell culture

Primary mutant and wild-type MEFs were isolated at embryonic day 13.5 from *Bbs6*-null mice (Ross et al., 2005; Hernandez-Hernandez et al., 2013). The head and red organs were removed, and the remaining tissues were trypsinized and dissociated five times with a syringe, followed by the dissociation with a 25-μm needle for five times. Cells were transferred into flasks and incubated in DMEM/F-12 (Thermo Fisher, 31331093) supplemented with 1% Penicillin–Streptomycin–Glutamine (Thermo Fisher, 10378016) and 10% fetal bovine serum (Thermo Fisher, 10270106). After reaching confluency, cells were immortalized according to the 3T3 immortalization protocol (Reznikoff et al., 1973) and further cultured with constant passaging numbers. Cells were regularly genotyped as previously described (Ross et al., 2005). *Bbs6* knockout was confirmed by genotyping, as shown in Supplementary Figure S3H.

3T3-immortalized *Bbs8* wild-type and knockout MEFs were a kind gift from Dagmar Wachten's laboratory (Institute of Innate Immunity, Bonn), isolated and cultivated in the same way as *Bbs6* wild-type and knockout MEFs. *Bbs8* knockout was confirmed by genotyping, as shown in Supplementary Figure S3I.

For serum starvation experiments, MEFs were cultured for 24–48 h in OptiMEM (Thermo Fisher, 11058021) prior to harvest. For pulldown assays, HEK293T cells were obtained from American Type Culture Collection (ATCC) and cultured in DMEM–GlutaMAX (Thermo Fisher, 31966047) supplemented with 10% fetal bovine serum and 1% Penicillin–Streptomycin–Glutamine. All cell lines were tested regularly for mycoplasma contamination.

### Transfections

Plasmid transfection in HEK293T cells was conducted using Genetrap transfection reagent (made at the National Eye Institute, NIH) as previously described (Patnaik et al., 2019). MEFs were transfected with Lipofectamine 2000 (Thermo Fisher, 11668030) according to the manufacturer's instructions and fixed or imaged after 48 h. In the case of serum starvation experiments, cells were transfected and, after 24 h, were serum-starved for another 24 h prior to fixation and experiments. Knockdown by siRNA was performed with Lipofectamine RNAiMax transfection reagent (Thermo Fisher, 13778150) according to the manufacturer's instructions. Mouse *siFascin-1* and *siNTC* were obtained from IDT as Trifecta Kits (mm.Ri.Fscn1) and validated by RT–qPCR. The working concentration of each siRNA was 10 nM. For *siFascin-1*, siRNAs 3′-GAGACUUCUGGGUACUAUCAUUCGAAA-5′ and 3′-GACGAUGAAACUGUAGCUCACCACACU-5′ were used in combination. Knockdown efficiency was validated, as shown in Supplementary Figure S3.

### RNA isolation and RT–qPCR

Total RNA was isolated from cells using TRIzol reagent (Thermo Fisher, 15596026) according to the manufacturer's instructions. RNA concentration and purity were measured using the NanoDrop™ 2000c Spectrophotometer (Thermo Fisher). Approximately 1000 ng of total RNA was reverse-transcribed into cDNA by the GoScript Probe 2-step RT–qPCR system (Promega, A5000). Respective cDNA quantity was determined by the StepOnePlus™ Real-Time PCR System (Applied Biosystems, 4376600) using SYBR Green (Platinum™ SYBR™ Green qPCR SuperMix-UDG, Thermo Fisher, 11733046) according to the manufacturer's recommendation. Cycling conditions were as follows: 95°C for 10 min followed by 40 cycles of 95°C for 15 sec and 60°C for 1 min. Specificity of the amplified product was determined by melt curve analysis. Relative target gene expression (fold change) was normalized to *Gapdh* or *Ywhaz* (only in *Bbs6* samples) and analyzed by 2^−ΔΔCT^ method. RT–qPCR primers are listed in Supplementary material.

### Live cell imaging

MEFs were seeded at low confluency in imaging chambers (ibidi, 80826) and transfected with mRFP-Lifeact and EGFP-FSCN-1 at 24 h post seeding. *Bbs6* wild-type and knockout MEFs were imaged at 48 h after transfection with a Leica SP8 confocal microscope with photomultipliers and a HyD detector (HC PL APO 63×/1.40 OIL, Leica) under the conditions of 37°C supplied with 5% CO_2_. Videos of *Bbs8* wild-type and knockout MEFs in an environmental chamber maintained at 37°C/5% CO_2_ were acquired on a Nikon A1R inverted laser scanning confocal microscope (Nikon Instruments) combined with a 60× Plan Fluore oil immersion objective (NA 1). Excitation wavelengths of 488 nm and 561 nm (diode lasers) were used. To follow their movement, cells co-expressing mRFP-Lifeact and EGFP-FSCN-1 were imaged every 5 sec for 30 timepoints. Videos were acquired either with the Leica LAS X (version 3.5.7.23225) or Nikon NIS-Elements (v4) imaging software.

### Filopodia measurement

Videos were processed with Fiji/ImageJ software (NIH) as tiff stacks, and the length of each filopodium per cell was measured with the FiloQuant plugin for Fiji as previously described (Jacquemet et al., 2019). The following parameters were used: threshold for cell edges (25), edge detection number of iterations for open (6), threshold for filopodia (25), filopodia minimum size (10), and contour measurement settings (number of iterations for close: 4; for erode: 2; for dilate: 2). Resulting filopodia lengths were used for further statistical analysis.

### Immunocytochemistry

Cells were seeded on glass coverslips at 24 h prior to transfections or treatments. Forty-eight hours after knockdown or serum starvation, cells were washed with sterile phosphate-buffered saline (PBS). According to the antibody requirements, methanol fixation (100% ice-cold methanol for 10 min on ice) or PFA fixation (4% PFA for 10 min at room temperature) was used. Following PFA fixation, quenching was performed with 50 mM NH_4_Cl for 10 min. Cells were permeabilized with PBSTx (PBS + 0.3% Triton X) for 15 min and blocked with Fishblock blocking buffer (0.1% ovalbumin, 0.5% fish gelatine in PBS, and 0.3% Triton X) for 1 h at room temperature. Primary antibodies were incubated in Fishblock overnight at 4°C. Antibodies used are listed in Supplementary material. Samples were washed three times for 10 min with PBSTx and incubated with corresponding secondary antibodies for 1 h at room temperature. Then, samples were washed again twice with PBSTx and once with PBS before mounting coverslips on glass slides. Images were taken at room temperature either by a Leica SP8 confocal microscope with photomultipliers and a HyD detector (HC PL APO 63×/1.40 OIL CS2, Leica) or by a Leica DM6000 microscope with a k5 sCMOS camera at 100× magnification/1.40 oil (Leica). Images showing filopodia were taken by a Zeiss LSM 900 with Airyscan 2 (63×/1.4 oil M27, Carl Zeiss Microscopy). Image processing, cilia length measurement, and fluorescence intensity quantification were all performed with Fiji/ImageJ software.

### Pulldown assays and western blotting

For interaction studies, HEK293T cells were co-transfected with EGFP-FSCN-1, mRFP-FSCN-1, EGFP-INVS, pCMV-MYC-BBS8, pCMV-MYC-BBS6, and/or empty vector using Genetrap reagent. After 48 h, cells were lysed in RIPA buffer (50 mM Tris–HCl, pH 8.0, 150 mM NaCl, 1% NP-40, 0.5% sodium deoxycholate, and 0.1% sodium dodecyl sulfate (SDS)) containing Halt™ Protease and Phosphatase Inhibitor Cocktail (100×, Thermo Fisher, 78442). Pulldown experiments for EGFP were performed with magnetic agarose beads (GFP-Trap-MA, ChromoTek) according to the manufacturer's instructions. Proteins were washed off the beads with 1× Laemmli loading buffer containing SDS, dithiothreitol (DTT), and β-mercaptoethanol at 95°C for 10 min. For co-immunoprecipitation of Fascin-1 and ubiquitin, MEFs were treated with 10 μM MG132 (Calbiochem, 474791) for 5 h before harvest, and lysates were prepared as described above. For each sample, 12.5 μl Dynabeads Protein-G (Fisher Scientific, 10003D) was washed with 500 μl PBSTx (0.01% Triton X) and incubated rotating for 4 h at 4°C with 6 μl Fascin antibody (mm, Invitrogen, MA5-11483) or mouse IgG control. Then, lysates were incubated rotating on antibody-coated beads overnight at 4°C. Beads were washed three times with PBSTx, and proteins were eluted with 1× Laemmli buffer without DTT and β-mercaptoethanol at 95°C for 10 min. Before loading on gel, DTT and β-mercaptoethanol were added again to avoid protein aggregates.

Proteins were separated on 10% polyacrylamide gels via SDS–polyacrylamide gel electrophoresis followed by western blotting. Proteins were transferred onto PVDF membranes (Immobilon^®^-FL PVDF membrane, Sigma, 05317) and blocked with AppliChem blocking buffer (0.2% AppliChem Blocking Reagent, 10 mM Tris–HCl, 150 mM NaCl, and 0.04% NaN_3_, pH 7.4) or 5% milk or bovine serum albumin according to the antibody requirements. Membranes were probed with antibodies overnight at 4°C, washed with Tris-buffered saline containing 0.1% Tween, and incubated with corresponding secondary antibodies for 1 h at room temperature. Membrane scanning was performed with the Odyssey Infrared Imaging System (Licor) at 680 or 800 nm. Densitometry analysis was performed with Fiji/ImageJ software, and the expression levels were normalized to the input or Gapdh expression level. Antibodies used for western blotting are listed in Supplementary material.

### Statistical analysis

Statistical analysis was performed using IBM SPSS 27 software. Parametric or non-parametric data distribution was determined using the Shapiro–Wilk test, and outliers were extracted. Parametric differences were determined using the *t*-test. Differences between two non-parametric groups were compared using a Mann–Whitney *U*/Wilcoxon signed-rank test. *P*-values of 0.05 and below were considered statistically significant. Statistical tests and number of repetitions are described in the legends. Boxplots show the median (middle line), edges of boxes are top and bottom quartiles (25%–75%), and whiskers represent the ranges for the upper 25% and the bottom 25% of data values. Outliers are shown as circles above and below whiskers. Bar plots show the mean ± standard errors.

## Supplementary Material

mjad022_Supplemental_Files
