# Supplementary material for: The actin-bundling protein Fascin-1 modulates ciliary signalling
Source: J Mol Cell Biol. 2023 Apr 4;15(4):mjad022. doi: 10.1093/jmcb/mjad022 (PMC10485897; doi:10.1093/jmcb/mjad022)
Supplement: mjad022_Supplemental_Files [file mjad022_supplemental_files.zip › JMCB-2022-0046_Supplementary Material.pdf]

Supplementary data

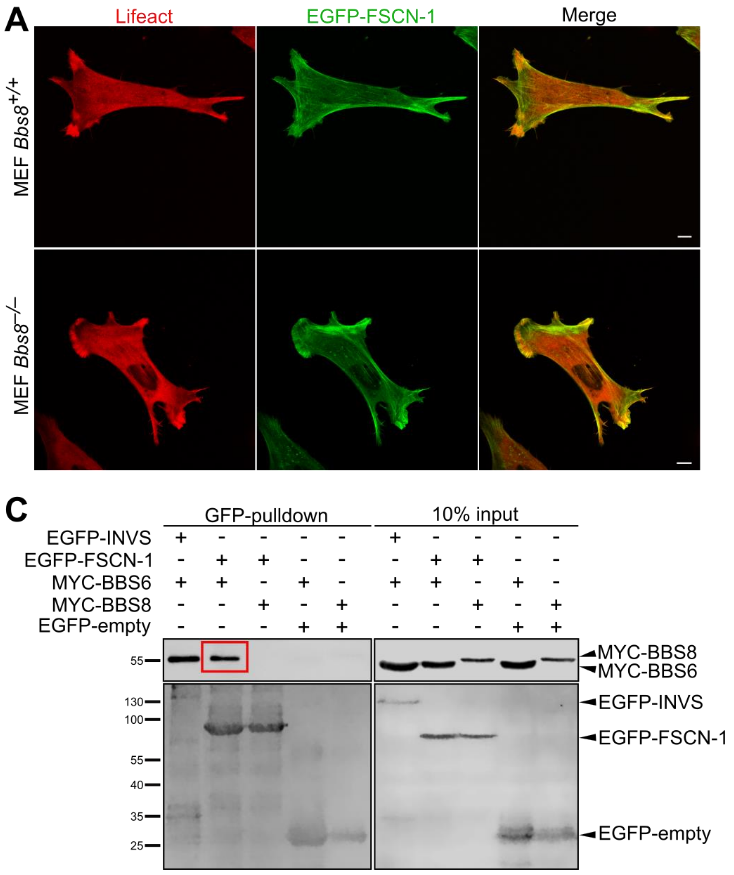

Supplementary Figure S1: BBS8 does not affect filopodia dynamics.

A Live cell imaging of *Bbs8*$^{+/+}$ and *Bbs8*$^{-/-}$ MEFs overexpressing mRFP-Lifeact for the actin cytoskeleton and EGFP-FSCN-1 as a filopodia marker. Images represent one timepoint out of 30, videos are shown in supplementary videos S3 and S4. **B** Average filopodia length via FiloQuant analysis shows no significant difference in filopodia length upon *Bbs8* knockout (Mann-Whitney-U test, $P = 0.74$). N(*Bbs8*$^{+/+}$)=18, N(*Bbs8*$^{-/-}$)=16. Experiments were repeated twice. **C** Interaction study between Fascin-1 (FSCN-1) and BBS6/BBS8. GFP pulldowns were performed 48 h after overexpression of EGFP-FSCN-1 and MYC-BBS6 or MYC-BBS8 in HEK293T cells. The interaction between EGFP-INVS (Inversin) and MYC-BBS6 was used as positive control, EGFP-empty served as negative control. MYC-BBS8 did not form a complex with EGFP-FSCN-1. Red box shows positive interaction between EGFP-FSCN-1 and MYC-BBS6 as shown in Figure 1.

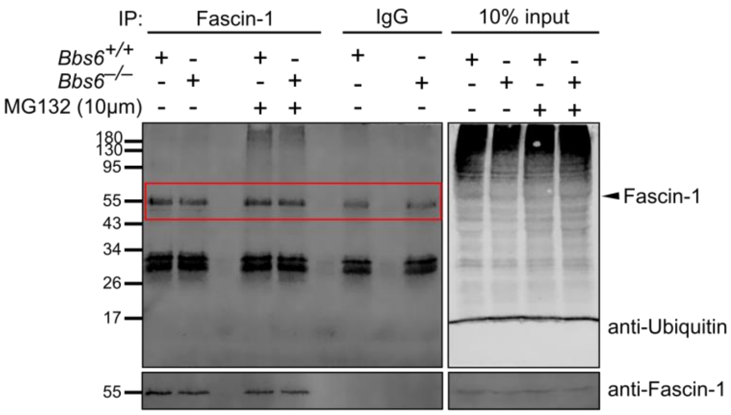

Supplementary Figure S2: Bbs6 does not affect ubiquitination of Fascin-1.

Immunoprecipitation of endogenous Fascin-1 of *Bbs6*$^{+/+}$ and *Bbs6*$^{-/-}$ MEFs with or without treatment of MG132 (5 h). Probing the membranes with Ubiquitin antibody reveals no differences between level of ubiquitinated Fascin-1 (red box) in *Bbs6* wildtype or knockout cells. N=2.

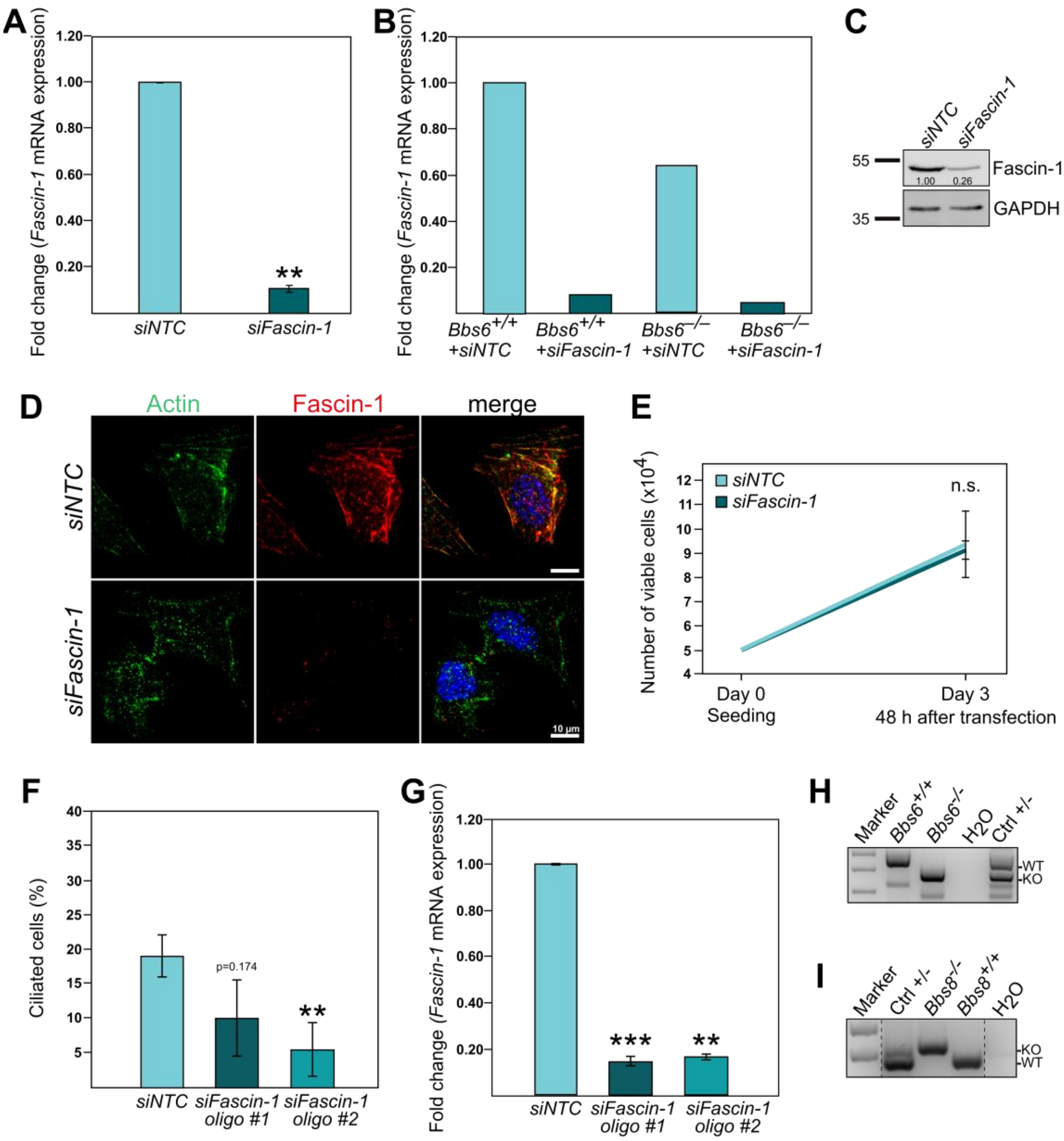

Supplementary Figure S3: Validation of mouse embryonic fibroblast models.

A mRNA level of *Fascin-1* in MEFs 48 h after knockdown measured by RT-qPCR. mRNA expression of *Fascin-1* is significantly reduced to ~11% after knockdown in comparison to *siNTC*. (Mann-Whitney-U, $P < 0.01$; N=3). Two siRNA oligos were used in combination to successfully silence *Fascin-1*. **B** mRNA level of *Fascin-1* in *Bbs6* wildtype and knockout MEFs 48 h after knockdown measured by RT-qPCR shows a successful reduction in mRNA of *Fascin-1* in both cell lines. **C** Protein expression of Fascin-1 in MEFs validated by western blot is reduced to 26% after knockdown of *Fascin-1*. Numbers show AUC for protein bands in relation to Gapdh and normalised to *siNTC* (fold change). **D** Visualisation of Fascin-1 (red) via endogenous immunofluorescence staining in MEFs reveals less signal for Fascin-1 after *Fascin-1* knockdown. **E** Proliferation assay in *Fascin-1* depleted cells. Counting of viable cell numbers 48 h after transfection with either *siNTC* or *siFascin-1* reveals no significant difference in cell numbers, indicating no effect of *Fascin-1* depletion on proliferation and cell cycle (Student's t-test; $P > 0.05$). **F** The ciliary phenotype upon *Fascin-1*

depletion can be recapitulated with two different siRNA oligos. Transfection with both oligos lead to reduced ciliary numbers (Student's t-test). **G** Validation of the efficiency for both siRNA oligos individually in silencing *Fascin-1* mRNA levels via RT-qPCR (Student's t-test, N=3). n.s. $P > 0.05$, * $P < 0.05$, ** $P < 0.01$, *** $P < 0.001$. **H+I** Genotyping of *Bbs6* (**H**) and *Bbs8* (**I**) MEFs according to Ross et al., 2005 and Tadenev et al., 2011. Heterozygous (Ctrl +/-) samples were used to define band sizes for WT (wildtype) and KO (knockout), H_2O as negative control.

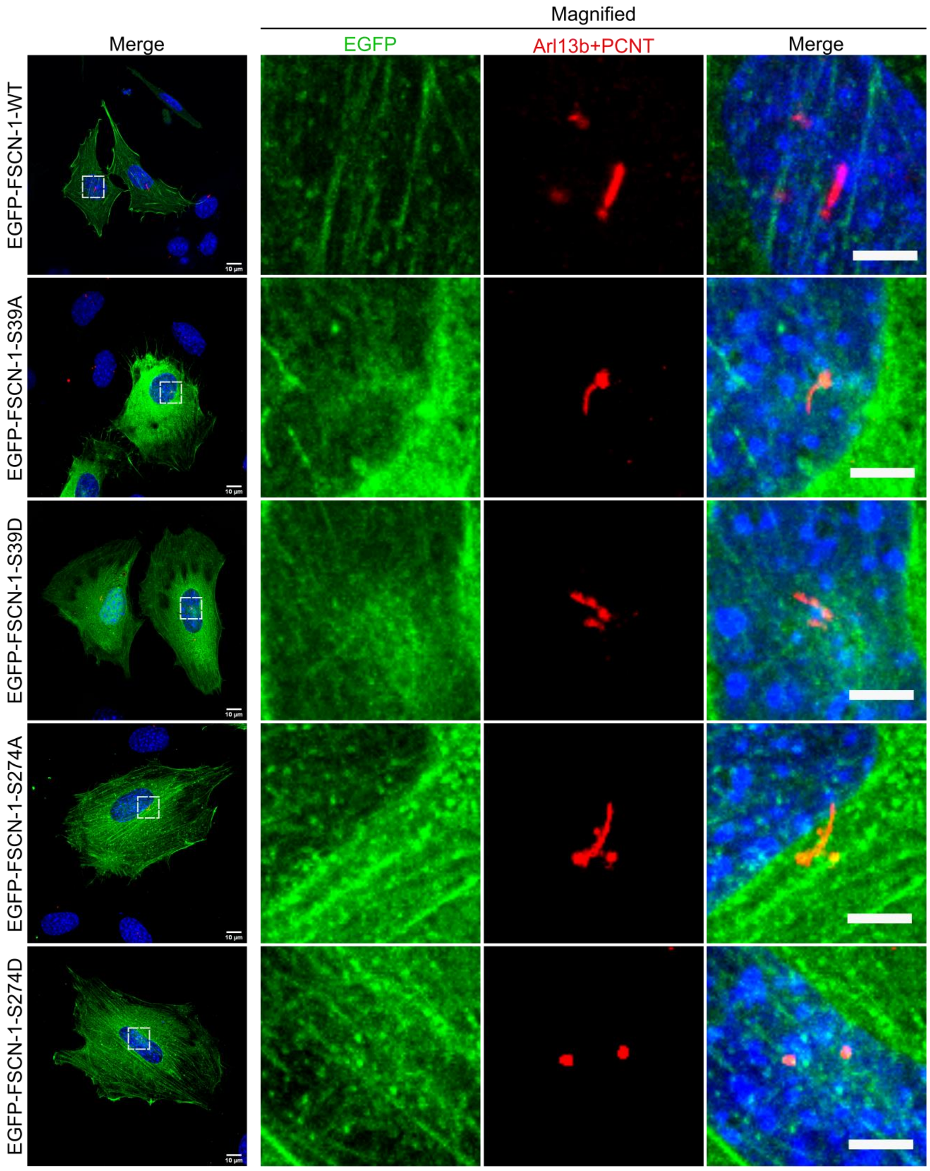

Supplementary Figure S4: Localisation of EGFP-tagged Fascin-1 mutations in MEFs.

Overexpression of EGFP-tagged FSCN-1 variants (green) and costaining with cilia markers PCNT (basal body) and Arl13b (cilia membrane; both red) 48 h after transfection in serum-depleted MEFs shows no improvement of ciliary localisation of the different mutations. Scale bar, 10 µm; Scale bar magnified images, 3 µm.

Supplementary Video S1: Live cell imaging of Bbs6$^{+/+}$ MEFs.

Live cell imaging of Lifeact (actin cytoskeleton, red) and EGFP-FSCN1 (filopodia, green) in *Bbs6* wildtype (*Bbs6*$^{+/+}$) mouse embryonic fibroblasts (MEFs), 48 h after transfection. Videos were taken every 5 sec for 30 timepoints and edited with Fiji. Scale bar: 10 µm.

Supplementary Video S2: Live cell imaging of Bbs6$^{–/–}$ MEFs.

Live cell imaging of Lifeact (actin cytoskeleton, red) and EGFP-FSCN1 (filopodia, green) in *Bbs6* knockout (*Bbs6*$^{–/–}$) mouse embryonic fibroblasts (MEFs), 48 h after transfection. Videos were taken every 5 sec for 30 timepoints and edited with Fiji. Scale bar: 10 µm.

Supplementary Video S3: Live cell imaging of Bbs8$^{+/+}$ MEFs.

Live cell imaging of Lifeact (actin cytoskeleton, red) and EGFP-FSCN1 (filopodia, green) in *Bbs8* wildtype (*Bbs8*$^{+/+}$) mouse embryonic fibroblasts (MEFs), 48 h after transfection. Videos were taken every 5 sec for 30 timepoints and edited with Fiji. Scale bar: 10 µm.

Supplementary Video S4: Live cell imaging of Bbs8$^{–/–}$ MEFs.

Live cell imaging of Lifeact (actin cytoskeleton, red) and EGFP-FSCN1 (filopodia, green) in *Bbs8* knockout (*Bbs8*$^{–/–}$) mouse embryonic fibroblasts (MEFs), 48 h after transfection. Videos were taken every 5 sec for 30 timepoints and edited with Fiji. Scale bar: 10 µm.

Supplementary Materials and Methods

Antibodies

For immunofluorescence, primary antibodies were used as follows: anti-FSCN-1 (mm, 1:50; Invitrogen MA5-11483), anti-Arl13b (Rb, 1:800; Proteintech 17711-1-AP; mm, 1:200; Abcam N295B/66), anti-Actin (Rb, 1:200; Sigma A2066), anti-EGFP Living colors (Rb, 1:200; Takara Bio), anti-PCNT (1:500; Abcam Ab4448), anti-β-catenin (Rb, 1:200; Cell Signaling D10A8), anti-acetylated-β-catenin Lys49 (Rb, 1:150; Cell Signaling D7C2), anti-c-Jun (Rb, 1:100, Cell Signaling 9165), anti-Cyclin D1 (Rb, 1:150; Cell Signaling E3P5S).

Secondary antibodies used for immunofluorescence were DAPI (1:8000, Carl Roth 6843), Phalloidin TRITC (1:400, Sigma P1951), Phalloidin 647 (1:40, Cell Signal 8940), anti-mouse 488 (1:400, Invitrogen A21202), anti-mouse 555 (1:400, Invitrogen A31570), anti-rabbit 488 (1:400, Invitrogen A11034), anti-rabbit 555 (1:400, Invitrogen A21429).

For western blotting, the following antibodies were used: anti-Myc (mm, 1:1000; BD Biosciences 611013), anti-GFP (Rb, 1:1000; Chromotek Pabg1-10), anti-RFP (Rb, 1:7000; Thermofisher R10367), anti-FSCN-1 (mm, 1:1000; Invitrogen MA5-11483), anti-Gapdh (mm, 1:2000; Cell Signaling 97166), anti-Actin (Rb, 1:1000; Sigma A2066), anti-Cyclin D1 (Rb, 1:1000; Cell Signaling E3P5S), anti-GSK3β (Rb, 1:1000; Abcam Ab15580), anti-β-catenin (Rb, 1:1000; Cell Signaling D10A8), anti-acetylated-β-catenin Lys49 (Rb, 1:1000; Cell Signaling D7C2), anti-Axin2 (Rb, 1:600; Proteintech 20540-1-AP), anti-Dvl2 (Rb, 1:1000; Cell Signaling 3224), anti-Lrp6 (Rb, 1:1000; Cell Signaling 3395), anti-Arl13b (Rb, 1:1000; Proteintech 17711-1-AP), anti-Gt335 (mm, 1:1000; Adipogen AG-20B-0020-C100), anti-Inversin (Rb, 1:1000; Proteintech 10585-1-AP), anti-Ubiquitin (Rb, 1:500; Sigma-Aldrich 07-375)

Secondary antibodies for western blotting were anti-rabbit 680nm (1:10,000, LI-COR Biosciences 925-68073), anti-rabbit 800nm (1:10,000, LI-COR Biosciences 926-32211), anti-mouse 680nm (1:10,000, LI-COR Biosciences 925-68072), anti-mouse 800nm (1:10,000, LI-COR Biosciences 925-32212).

Primers RT-qPCR

**Gene**	**Forward**	**Reverse**
mGapdh	AATGGTGAAGGTCGGTGTGAA	AGGTCAATGAAGGGGTCGTTG
mYwhaz	TCTTGATCCCCAATGCTTCG	AATGCTTCTTGGTATGCTTGC
mBbs6	GTGTGCTCTGCAAGATTTGG	AAGACGTGCATTGCTGTTTG
mInversin	TCGCTGATGGAAACCTAACG	AAGGAGATGGACAATCTGTGC
mArl13b	CTGGGATGTTCAGTCTGATGG	TCTCCTTGGATTCCCTTTGC
mFascin-1	GTTGGAATTCAATGACGGCG	ACCTTGAGAGCCACCTTATTG
mCyclind1	TGCCATCCATGCGGAAA	AGCGGGAAGAACTCCTCTTC
mmyc	GCTGTTTGAAGGCTGGATTTC	GATGAAATAGGGCTGTACGGAG
mLrp5	GGGTCCACAAGGTCAAGGC	GCACCCTCCATTTCCATCC
mLrp6	GCCCACTACTCCCTGAATGCTG	TGTGGATAGGAAGGATGATGTCAGG

mDvl2	GGCTTGTGTCGTCAGATACC	TTTCATGGCTGCTGGATAC
mDvl3	CCGATGAGGATGATTCCACC	TGAGGCACTGCTCTGTTCTG
mAxin2	GAGTAGCGCCGTGTTAGTGACT	CCAGGAAAGTCCGGAAGAGGTATG
mβ-catenin	GTGCAATTCCTGAGCTGACA	CTTAAAGATGGCCAGCAAGC
mLef1	GTCCCTTTCTCCACCCATC	AAGTGCTCGTCGCTGTAG
mβ-actin	CACAGCTGAGAGGGAAATCGTGC	GATCTTGATCTTCATGGTGCTAGG
